# T-type calcium channels regulate medulloblastoma and can be targeted for therapy

**DOI:** 10.1007/s11060-025-04967-5

**Published:** 2025-02-17

**Authors:** Collin J. Dube, Michelle Lai, Ying Zhang, Shekhar Saha, Ulas Yener, Farina Hanif, Kadie Hudson, Myron K. Gibert, Pawel Marcinkiewicz, Yunan Sun, Tanvika Vegiraju, Esther Xu, Aditya Sorot, Rosa I. Gallagher, Julia D. Wulfkuhle, Ashley Vernon, Lily Dell’Olio, Rajitha Anbu, Elizabeth Mulcahy, Benjamin Kefas, Fadila Guessous, Emanuel F. Petricoin, Roger Abounader

**Affiliations:** 1 Department of Microbiology, Immunology & Cancer Biology, University of Virginia, Charlottesville, VA 22908 USA; 2 Department of Neurosurgery, Stanford University School of Medicine, Stanford, CA USA; 3 Department of Biochemistry, Dow International Medical College, Dow University of Health Sciences, Karachi, 75270 Pakistan; 4 Department of Pharmacy, University of Virginia, Charlottesville, VA 22908 USA; 5 Laboratory of Onco-Pathology, Biology and Cancer Environment, Faculty of Medicine, University of Sciences and Health, Mohammed, Casablanca, VI Morocco; 6 George Mason University Center for Applied Proteomics and Molecular Medicine, Manassas, VA 20155 USA; 7 Department of Neurology, University of Virginia, Charlottesville, VA 22908 USA; 8 University of Virginia Comprehensive Cancer Center, Charlottesville, VA 22908 USA; 9 Center for RNA Science and Medicine, University of Virginia, PO Box 800168, Charlottesville, VA 22908 USA

**Keywords:** Medulloblastoma, T-type calcium channels, Mibefradil

## Abstract

**Purpose:**

The goal of this study was to investigate the role and therapeutic targeting of T-type calcium channels in medulloblastoma, a common and deadly pediatric brain tumor that arises in the cerebellum.

**Methods:**

T-type calcium channel expression was assessed in publicly available bulk and single cell RNA-seq datasets. The effects of T-type calcium channel blocker mibefradil on cell growth, death and invasion were assessed with cell counting, alamar blue, trypan blue and transwell assays. Proteomic-based drug target and signaling pathway mapping was performed with Reverse Phase Protein Arrays (RPPA). Co-expression modules of single cell RNA-seq data were generated using high dimensional weighted gene co-expression network analysis (hdWGCNA). Orthotopic xenografts were used for therapeutic studies with the T-Type calcium channel blocker mibefradil.

**Results:**

T-type calcium channels were upregulated in more than 30% of medulloblastoma tumors and patients with high expression associated with a worse prognosis. T-type calcium channels had variable expression across all the subgroups of medulloblastoma at the bulk RNA-seq and single-cell RNA-seq level. Mibefradil treatment or siRNA mediated silencing of T-type calcium channels inhibited tumor cell growth, viability and invasion. RPPA-based protein/phosphoprotein signal pathway activation mapping of T-type calcium channel inhibition and single cell hdWGCNA identified altered cancer signaling pathways. Oral administration of mibefradil inhibited medulloblastoma xenograft growth and prolonged animal survival.

**Conclusion:**

Our results represent a first comprehensive multi-omic characterization of T-type calcium channels in medulloblastoma and provide preclinical data for repurposing mibefradil as a treatment strategy for these relatively common pediatric brain tumors.

**Supplementary Information:**

The online version contains supplementary material available at 10.1007/s11060-025-04967-5.

## Introduction

Medulloblastoma is a CNS tumor that arises in the cerebellum, primarily in pediatric patients [1, 2]. The 5-year survival rate for pediatric medulloblastoma patients is ∼ 70% [1]. Patients often experience permanent neurological deficits such as reduced motor and cognitive functions as a result of radiation and chemotherapy [3, 4]. Medulloblastoma is classified into four molecular subgroups: SHH, WNT, Group 3 and Group 4 [5–7]. SHH and WNT have a better prognosis than Group 3 and Group 4. Current treatment options for medulloblastoma include surgical resection and radio and chemotherapy but patients frequently experience permanent neurological deficits such as reduced motor and cognitive functions, reducing their overall quality of life [8–10].

T-type calcium channels are voltage gated channels that are active around resting membrane potential. They channel calcium from the extracellular space into the cell. Calcium signaling regulates numerous important cellular processes such as proliferation, migration, invasion and apoptosis [11, 12]. T-type calcium channels are deregulated in some cancers including colon carcinoma, breast cancer and gliomas [13–16]. Inhibition of T-type calcium channels with the selective inhibitor mibefradil has shown significant anti-tumor effects in gliomas in preclinical [13, 17, 18]. These studies formed the foundation for a Phase 1 clinical trial of mibefradil with temozolomide in recurrent glioma patients. The trial showed that mibefradil was safe with minimal side effects and elicited responses in select patients [19]. However, a comprehensive study of the expressions, functions and therapeutic targeting of T-type calcium channels in medulloblastoma has, to our best knowledge, not been published to date.

This study aimed to elucidate the role of T-type calcium channels in medulloblastoma using a multi-omic approach incorporating transcriptomic, proteomic/phosphoproteomics, coupled to functional analyses, and experimental therapy. We hypothesized that T-type calcium channels act as oncogenes to promote medulloblastoma malignancy parameters. We analyzed publicly available data and found that T-type calcium channels are upregulated in medulloblastoma tumors and that expression correlates with patient survival. Inhibition of T-type calcium channels with mibefradil significantly reduced medulloblastoma cell growth and invasion. Additionally, mibefradil significantly reduced tumor volume in vivo and prolonged animal survival. Mechanistically, we demonstrated that inhibition of T-type calcium channels regulates numerous cellular pathways such as cell proliferation and apoptosis. Also, T-type calcium channels are co-expressed in gene modules regulating numerous cellular pathways in medulloblastoma tumors. This is the first study elucidating the roles and therapeutic targeting of T-type calcium channels in medulloblastoma.

## Methods

### Cell lines

Three medulloblastoma cell lines were used in this study. PFSK, DAOY and ONS-76 were grown in RPMI-1640 media supplemented with 10% FBS. All cells were cultured in media in a 37 °C incubator with 5% CO_2_ and 20% O_2_. All cell lines underwent testing for species identity and mycoplasma infection using Mycoplasma Detection Kit-QuickTest.

### Pediatric brain tumor atlas analysis

Pediatric Brain Tumor Atlas data was downloaded from PedCbioPortal [20, 21]. Medulloblastoma tumors were subsetted for analysis of expression of the T-type calcium channels. Oncoplots of mutational status and survival curves of T-type calcium channels were generated using pedcbioportal.com with RNA-expression z-scores set to 2.0.

### Single-cell RNA seq analysis

The counts matrix from a published dataset (GSE155446) [22] was analyzed using the Seurat (v5) package in R (v3.4.1). The dataset underwent standard preprocessing using Seurat. Cell type categorization was conducted using the annotations published alongside GSE155446. T-type calcium channels were subsetted for > 0 for plotting the violin plots.

### hdWGCNA

Identification of co-expressed genes within tumor cells was carried out using hdWGCNA (v0.2.23) [23]. Meta cells were constructed using harmony dimensionality reduction. Modules were identified using the ConstructNetwork function below the optimal soft threshold. To identify module feature genes, ModuleEigenees function was used followed by computing of the module connectivity to identify hub genes with ModuleConnectivity functions. FindDME function was used to identify differentially expressed module eigengenes between cells that express Cav3.1 or Cav3.2 vs. cells that had no expression. Pathway enrichment analysis of gene modules was performed using enrichR (v3.2).

### Transfection

Cells were transfected with either 30 nM of a scrambled negative control (Thermofisher #AM4635) or 30 nM of a siRNA for Cav3.2 (ThermoFisher #AM16708) using RNAiMax reagent. Cells were harvested 48 h post-transfection for various analyses, including RNA isolation, cDNA synthesis, qPCR, or functional assays.

### Cell death and proliferation assays

Medulloblastoma cell lines were either drug treated (mibefradil 1–10 µM) or transfected with siRNA for Cav3.2. Cell death was assessed by trypan blue assay. The Alamar Blue assay which measures cell viability was utilized according to the manufacturer’s instruction. Cell proliferation was assessed by cell counting for five days as previously described [13, 24, 25]. All experiments were performed three times.

### Invasion assay

Medulloblastoma cell lines were either drug treated (mibefradil 10 µM) or transfected with siRNA for Cav3.2. After 72 h cells were seeded at 100,000 cells per chamber in 0.01% FBS media in collagen IV coated chambers. The chambers were incubated in complete media with 10% FBS in 24 well plate in triplicates. After 8 h of incubation the chambers were gently rinsed with PBS and stained with 0.1% crystal violet solution in 20% methanol. Chambers were imaged on Evos XL Core microscope. The images were quantified with ImageJ software [26].

### Reverse phase protein arrays

Proteomic screening was performed by Reverse Phase Protein Array (RPPA) as previously described [27–29]0.100 protein and phosphoprotein analytes were chosen for analysis based on their involvement in key aspects of tumor biology. All antibodies were validated as previously described [27–29]. For proteomic screening, ONS-76 and DAOY cells were treated with vehicle control or mibefradil 10 µM.

### Immunoblotting

Immunoblotting was performed as previously described [30]. Antibodies used were Ki67 (ab16667), BAK (ab32371), BAD (ab32445), CDK2 (SC-163), HSP90 (SC-13119) and GAPDH (SC-166545).

### In vivo experiments

Medulloblastoma DAOY cells (3 × 10^5^) were stereotactically implanted into the cerebellum of immunodeficient mice (*n* = 7 per treatment group). Seven weeks after tumor implantation, the brains were scanned with magnetic resonance imaging on a 7 Tesla Bruker/Siemens ClinScan small animal MRI. After confirmation of tumor presence, the animals were randomized and treated with control (H_2_O) or mibefradil (24 mg/kg) by oral gavage every 6 h once a day for four days. Mice were treated for two cycles (Days 49–52, 55–58). Brains were scanned with magnetic resonance imaging (MRI) following treatment cycles. Tumor volumes were quantified using OsiriX Lite software. For survival studies mice were monitored carefully and sacrificed when they displayed symptoms of tumor distress including lethargy and head tilt.

### Statistical analyses

Comparisons between means of samples were performed using Students t-tests and one-way ANOVA. All quantitative results are shown as means ± SEM. Molecular experiment tests, and computational experiment tests were performed using RStudio.

## Results

### T-type calcium channels are deregulated in medulloblastoma patient samples and expression correlates with survival

**Fig. 1:**
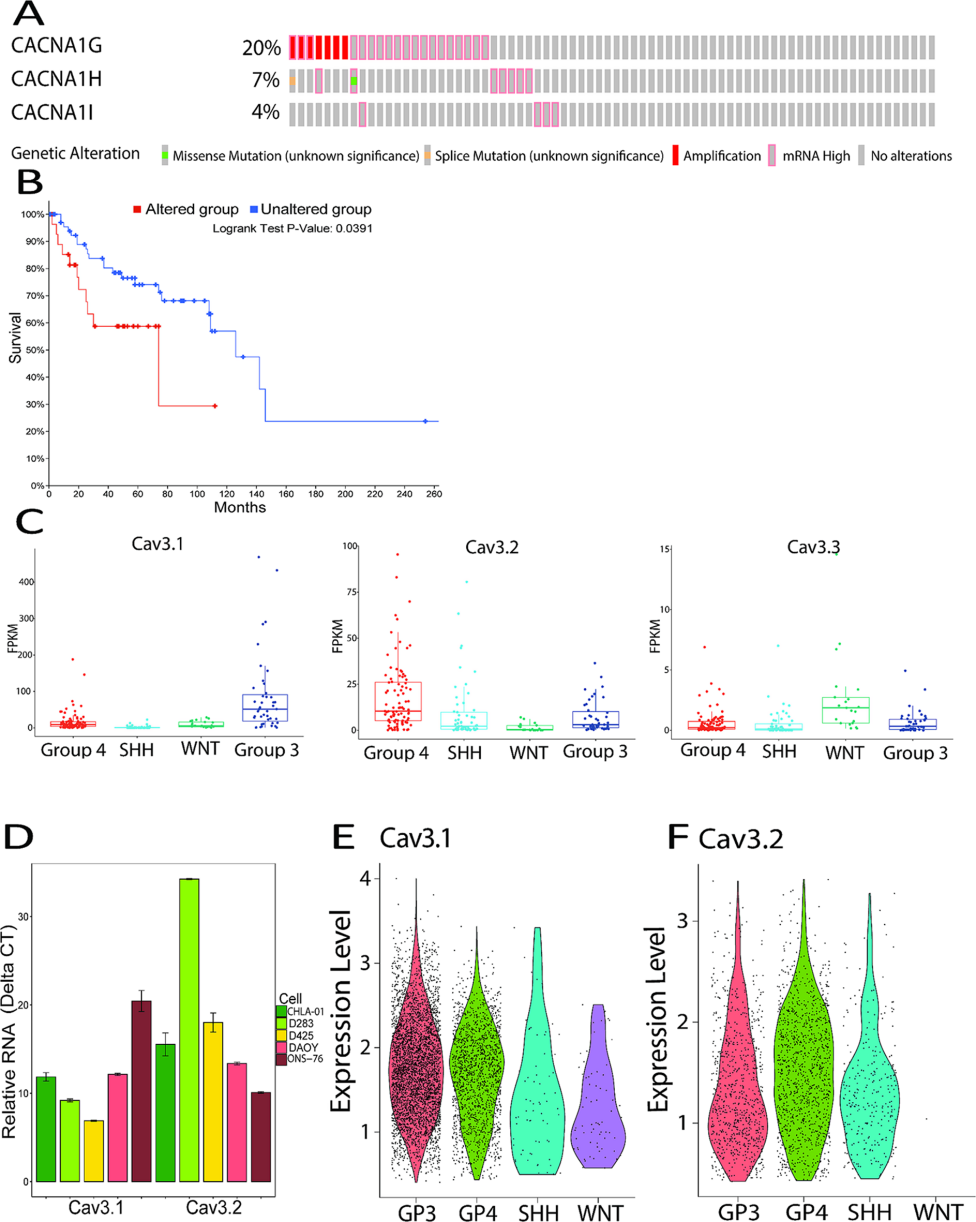
T-type calcium channels are dysregulated in medulloblastoma. (**A**) Genetic dysregulation of T-type calcium channels in medulloblastoma samples of the Pediatric Brain Tumor Atlas (PBTA). (**B**) Correlation of T-type calcium channel dysregulation with patient survival based on PBTA analysis. (**C**) Analysis of T-type calcium channel expression in subgroups from the Pediatric Brain Tumor Atlas (PBTA). (**D**) Relative expression Cav3.1 and Cav3.2 expression in medulloblastoma cell lines via RT-qPCR. (**E** & **F**) Violin plot showing expression of Cav3.1 and Cav3.2 in the different medulloblastoma subtypes in scRNA-seq data

To determine expression of T-type calcium channels in medulloblastoma, we analyzed 121 medulloblastoma samples in the Pediatric Brain Tumor Atlas (PBTA) [20] and identified T-type calcium channels as amplified or upregulated in greater than 30% of patients (Fig. 1A). This ranks T-type calcium channels as one of the most frequently deregulated genes in medulloblastoma. Patients with alterations in T-type calcium channels demonstrated a trend toward worse survival compared to patients with no alteration of T-type calcium channels (Fig. 1B). We examined expression of T-type calcium channels across medulloblastoma subgroups in the Pediatric Brain Tumor Atlas and found high expression of T-type calcium channels across multiple subgroups (Fig. 1C). We also examined expression of T-type calcium channels in medulloblastoma cell lines and found high expression of Cav3.2 in medulloblastoma cell lines (Fig. 1D). We examined expression of T-type calcium channels in publicly available single cell RNA-seq datasets and found that expression of Cav3.1 across all subtypes and Cav3.2 expression in SHH, Group 3 and Group 4 tumors (Fig. 1E & F) [22]. Cav3.3 was examined in the dataset but there weren’t enough positive cells to visualize them, so we focused primarily on Cav3.1 and Cav3.2. These data demonstrate that T-type calcium channels are upregulated in human medulloblastoma tumors and cell lines.

### Single cell high dimensional weighted gene co-expression network analysis (hdWGCNA) provides additional insights into the roles of T-type calcium channels in medulloblastoma

To gain further insight into the role of T-type calcium channels in medulloblastoma we performed single cell high dimensional weighted gene co-expression network analysis (hdWGCNA) on publicly available single-cell RNA seq medulloblastoma tumors [22]. We focused on the malignant cells of each subgroup to identify the co-expression networks for each subgroup of medulloblastoma. We identified 6 unique co-expression modules for SHH, 8 co-expression modules for Group 3, 3 co-expression modules for Group 4 and didn’t have enough statistical power to identify modules of the WNT subgroup.

**Fig. 2:**
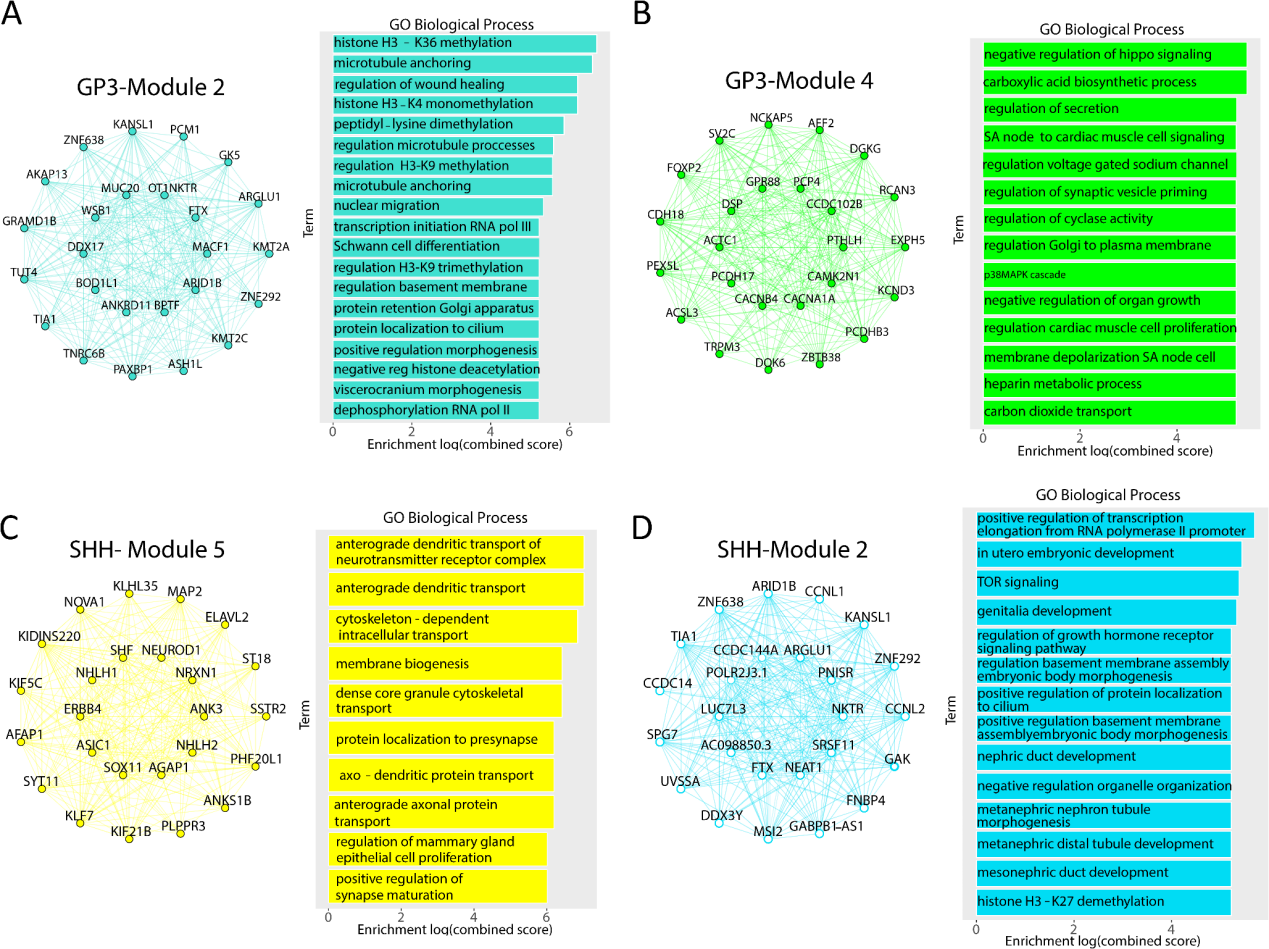
Cav3.1 and Cav3.2 are co-expressed with different pathways. (**A**) Network plot of top differentially expressed module for Cav3.1 positive cells in Group 3 tumors and associated biological processes. (**B**) Network plot of the top differentially expressed module for Cav3.2 positive cells in Group 3 tumors and associated biological processes. (**C**) Network plot of the top differentially expressed module for Cav3.1 positive cells in SHH tumors and associated biological processes. (**D**) Network plot of the top differentially expressed module for Cav3.2 positive cells in SHH tumors and associated biological processes

To gain insight into which modules the T-type calcium channels are impacting, we performed Differential Module Eigengene (DME) analysis between cells expressing one of the T-type calcium channels and cells which didn’t express them. We prioritized Cav3.1 and Cav3.2 since they are widely expressed in tumor cells, but we didn’t have enough Cav3.3 positive cells for follow-up analysis. Within the Group 3 tumors, Module 2 was most upregulated in Cav3.1 positive tumors and Module 4 was most upregulated in Cav3.2 positive tumors (Fig. 2A & B, Supplemental Fig. 1A-E). Interestingly the Cav3.1 and Cav3.2 positive cells exhibit opposite trends in their differential modules pointing toward different potential cell states between the Cav3.1 and Cav3.2 positive Group 3 tumors. We then performed Gene ontology pathway analysis to gain insight into what biological pathways were associated with the different modules. Module 2, which was upregulated in Cav3.1 positive tumors, was associated with biological processes related to histone methylation and microtubule movement (Fig. 2A). Module 4 which was upregulated in Cav3.2 positive tumors was associated with biological processes related to negative regulation of hippo signaling, regulation of secretion and regulation of synaptic vesicle pruning (Fig. 2B).

We next examined the DME of Cav3.1 and Cav3.2 positive tumor cells in the SHH modules. Module 5 was the most upregulated module in Cav3.1 positive tumors whereas Module 2 was the most upregulated module in Cav3.2 positive tumors (Fig. 2C & D, Supplemental Fig. 2A-E). We observed a similar trend in the SHH tumors where the Cav3.1 and Cav3.2 positive cells exhibited opposite regulation of their top modules. Analysis of biological pathways of Module 5 showed processes associated with neuronal functions such as synaptic localization and neurotransmitter transport (Fig. 2C). The analysis of the biological pathways of Module 2 revealed processes associated with signaling pathways such as TOR and regulation of growth hormone receptors (Fig. 2D). Analysis of Group 4 tumors identified three modules but no modules were differentially expressed between Cav3.1 and Cav3.2 positive tumor cells (Supplemental Fig. 3A-D). These data demonstrate that T-type calcium channels regulate numerous signaling pathways in the different subgroups of medulloblastoma.

### Blockade of T-type calcium channels with mibefradil inhibits cell growth, invasion and induces cell death

**Fig. 3:**
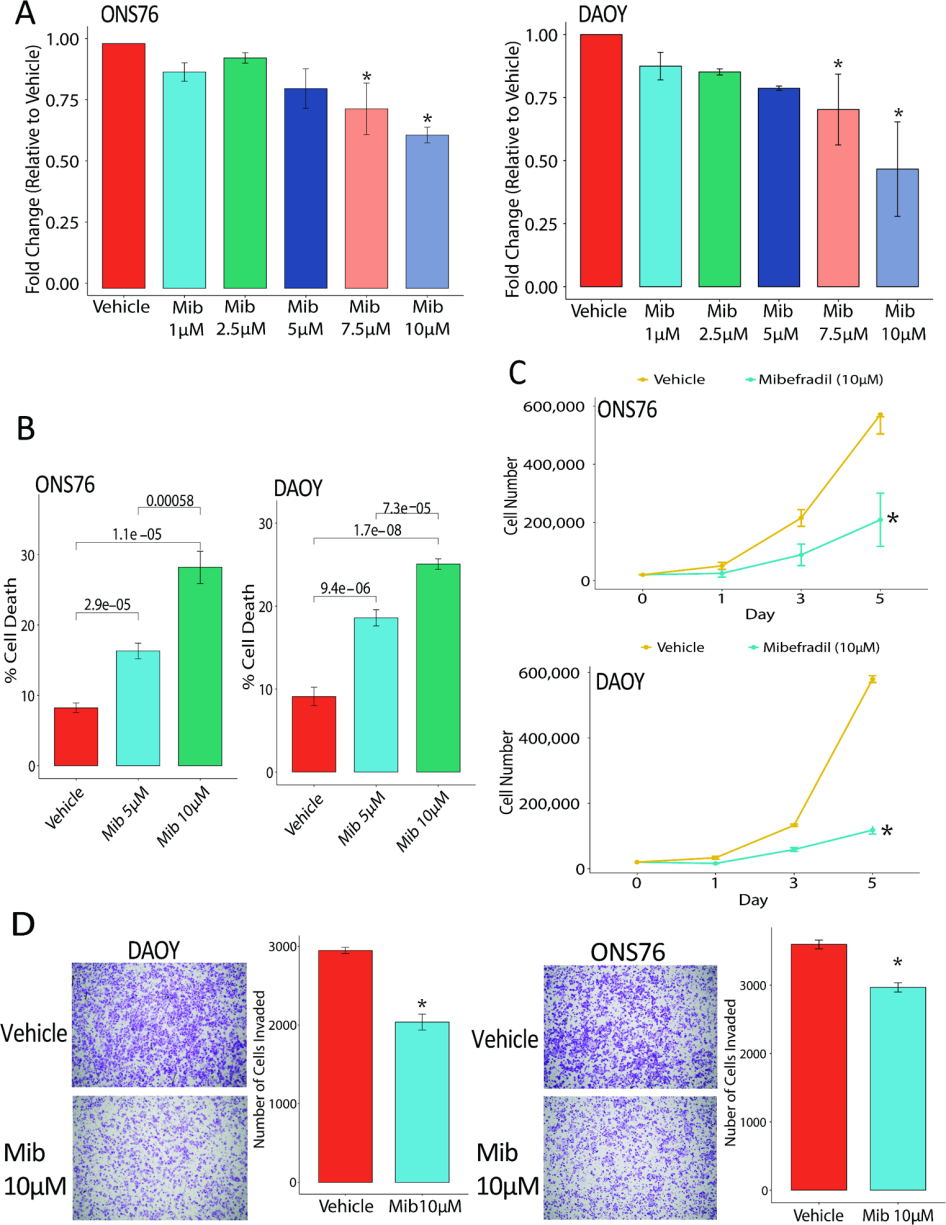
T-type calcium channel blocker mibefradil inhibits cell growth and induces cell death in medulloblastoma. (**A**) ONS-76 and DAOY cells were treated with vehicle or mibefradil (1–10 µM) and assessed cell viability changes by alamar blue 72 h later. (**B**) ONS-76 and DAOY cells were treated with vehicle or mibefradil and cell viability was assessed 48 h later by trypan blue cell counting. (**C**) Cell growth assay of vehicle and mibefradil treated cells (ONS-76. DAOY). (**D**) Representative images of invaded cells and quantification of invasive cell number in vehicle and mibefradil treated cells. *N* = 3 independent experiments for all assays. *p < 0.05

To examine the functional effects of blockage of T-type calcium channels on medulloblastoma, we utilized T-Type calcium channel blocker mibefradil to assess the effects of channel blockade on cell growth, invasion, proliferation and death. Medulloblastoma cell lines (PFSK, ONS-76, DAOY) were treated with vehicle or mibefradil (1–10 µM) and assessed for cell viability by alamar blue assay. The results demonstrate a dose dependent response to mibefradil inhibiting cell growth in all the medulloblastoma cell lines (Fig. 3A, Supplemental Fig. 4A). To determine the effects of mibefradil on cell death, we treated medulloblastoma cell lines with vehicle or mibefradil and performed trypan blue assay, which showed significant cell death upon mibefradil treatment (Fig. 3B, Supplemental Fig. 4B). To determine the effects of mibefradil on cell proliferation medulloblastoma cell lines were treated with mibefradil and counted over 5–7 days. Mibefradil significantly inhibited cell proliferation in all medulloblastoma cell lines (Fig. 3C, Supplemental 4 C). To determine if mibefradil impacts cell invasion we treated medulloblastoma cells as described above, then performed invasion assays. The results revealed that mibefradil significantly inhibited medulloblastoma invasion in all cell lines (Fig. 3D). Altogether these data demonstrate that mibefradil is a strong inhibitor of medulloblastoma cell growth, viability and invasion.

### Silencing of Cav3.2 inhibits cell growth and induces cell death

To ascertain that mibefradil-induced cell death and growth suppression are specifically due to T-type calcium channel inhibition, we silenced Cav3.2 in medulloblastoma cells using siRNA and assessed cell death and growth. Cells were transfected with either a scrambled siRNA or siRNA for Cav3.2. Knockdown was confirmed by qPCR which demonstrated 75% knockdown, as well at the protein level via Western Blot (Supplemental Fig. 4D). Knockdown of Cav3.2 significantly inhibited cell growth (Supplemental Fig. 4E). To assess how silencing of Cav3.2 impacts cell death, we utilized trypan blue cell counting. Silencing of Cav3.2 significantly increased cell death compared to scrambled control (Supplemental Fig. 4F). These data demonstrate that gene silencing of Cav3.2 significantly impacts medulloblastoma cell growth and death, comparable to mibefradil.

**Fig. 4:**
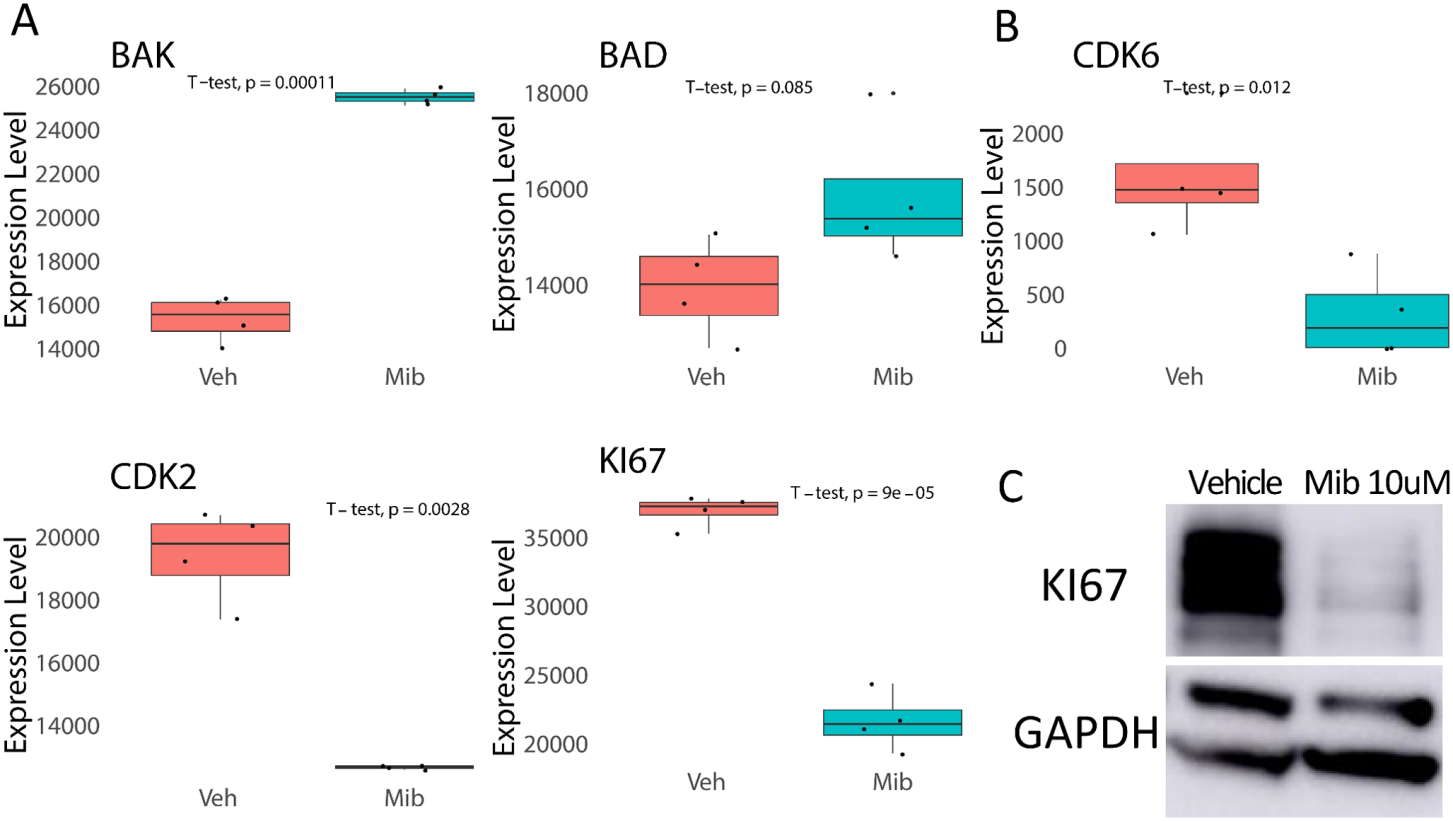
Mibefradil downregulates important apoptosis and growth regulating proteins. (**A**) Reverse phase protein array of mibefradil treated cells showed upregulation of BAK and BAD in medulloblastoma cells. (**B**) Reverse phase protein array of mibefradil treated cells showed downregulation of cell growth and cell cycle proteins such as CDK6, CDK2 and Ki67. (**C**) Western blot confirmation showing downregulation of Ki67 in mibefradil treated ONS-76 cells

### Inhibition of T-type calcium channels induces apoptosis pathways and inhibits cell proliferation pathways

To investigate the mechanism of action of T-type calcium channels we treated medulloblastoma cells with mibefradil or vehicle control and performed RPPA on the resultant lysates. Mibefradil treatment significantly induced widespread changes in medulloblastoma signaling (Supplemental Fig. 5A). There were significant changes in the induction of apoptosis signaling activation of BAK and BAD (Fig. 4A). Additionally, mibefradil treatment affected molecules associated with cell proliferation such as the decrease of CDK6, CDK2 and Ki67 (Fig. 4B). These results were verified by immunoblotting (Fig. 4C and Supplemental Fig. 5B). We also examined the overlap the RPPA and hdWGCNA which revealed important regulators of medulloblastoma tumor signaling with 6 targets overlapping in SHH (Ephrin A3, FOXM1, Histone H2A, Ki67, Survivin and Synaptophysin), 12 overlapping targets in Group 3 (EGFR, FoxM1, Glucocorticoid receptor, Histone H2A, Ncam1, p38 MAP kinase, PTEN, CDK6, Ki67, LC3B, Survivin, and Twist) and 3 overlapping targets in Group 4 tumors (FoxM1, Ki67 and Survivin). The above data show that mibefradil inhibition of T-type calcium channel regulates medulloblastoma cell signaling including apoptosis and cell proliferation associated molecular events.

### T-type calcium channel blockage inhibits in vivo tumor growth and prolongs animal survival

**Fig. 5:**
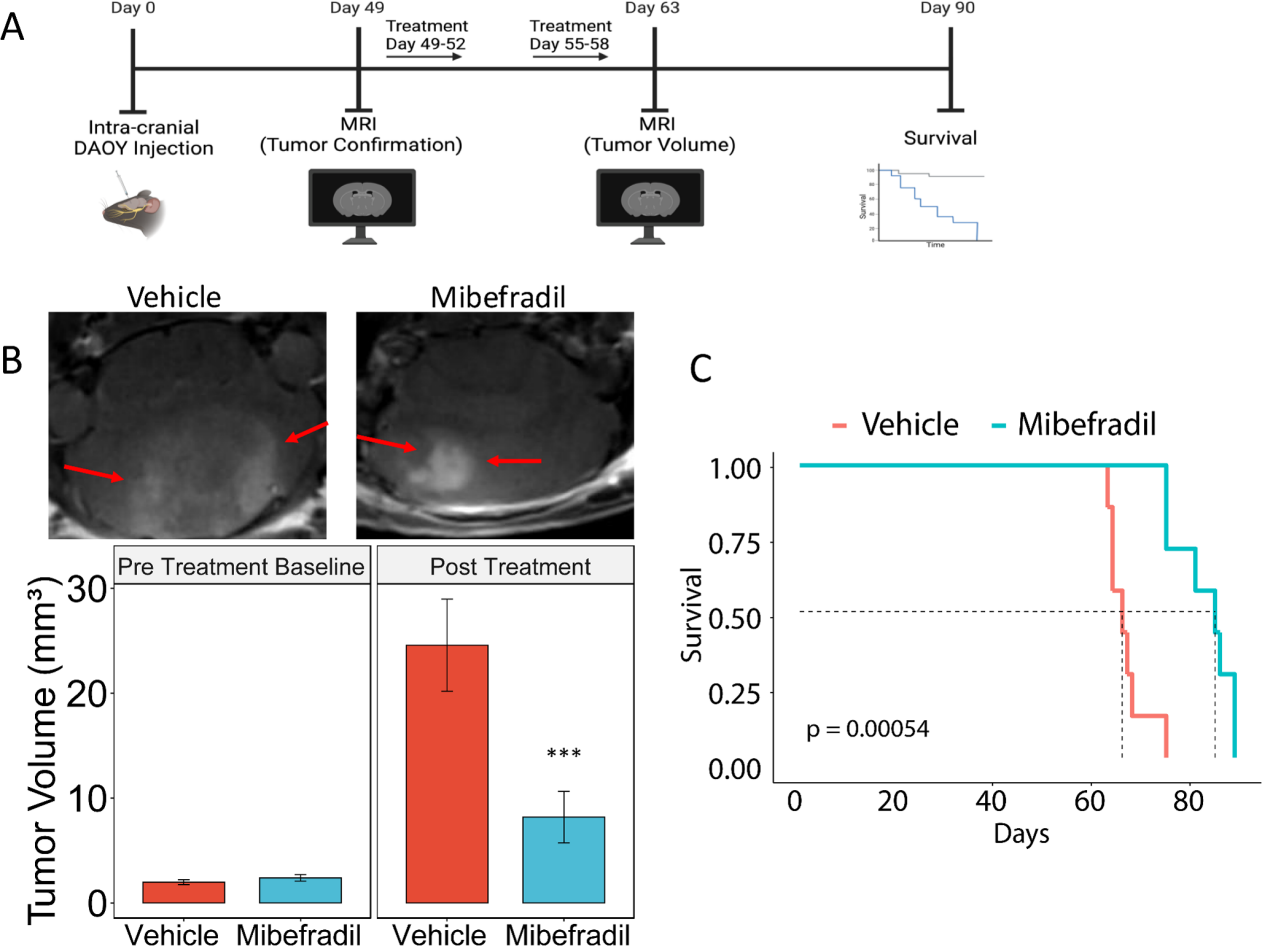
Mibefradil inhibits DAOY xenograft growth and prolongs survival. (**A**) Schematic of in vivo experimental design. (**B**) Representative MRIs of the tumor with quantification of tumor volume at the pretreatment time point as well as the post treatment timepoint for both Vehicle and Mibefradil-treated mice. (**C**) Kaplan-Meier survival curves of vehicle and mibefradil-treated mice. *N* = 7 mice per group. ***p < 0.001

To determine the effects of blockade of T-Type calcium channels with the FDA approved repurposed channel blocker mibefradil on tumor growth in vivo, we utilized medulloblastoma xenografts in immunodeficient mice. DAOY cells (3 × 10^5^) were stereotactically implanted in the cerebellum and tumor formation was monitored by MRI. Seven weeks after tumor implantation the mice were subjected to MRI. Pre treatment baseline measurements were recorded followed by treatment of either vehicle (H_2_O) or mibefradil (24 mg/kg body weight) which was administered per oral gavage every six hours for 4 days (Fig. 5A, B). The treatment plan was repeated three days later. MRI scans were performed two weeks after drug treatment and tumor volume was quantified. Animal survival was also assessed. Mibefradil significantly inhibited tumor growth (Fig. 5B). Additionally, mibefradil significantly prolonged survival compared to vehicle treated mice (Fig. 5C). These data show that inhibition of T-type calcium channels with mibefradil significantly reduces tumor volume and prolongs survival of mice, providing support for the use of mibefradil as a therapeutic drug in future clinical trials in medulloblastoma patients.

## Discussion

Calcium signaling regulates a plethora of cellular processes such as proliferation, migration, invasion and apoptosis. Calcium channels are deregulated in cancers, where they can serve as a therapeutic target. Mibefradil is an orally bioavailable blocker of T and L type calcium channels, marketed by Roche as Posicor for the treatment of hypertension and previously in clinical trials for cancer.

We demonstrated that T-type calcium channels are upregulated in medulloblastoma tumors and cell lines. Patients that exhibit alterations in T-type calcium channels had worse survival compared to patients with no alterations. Patients with alterations in T-type calcium channels typically exhibit a mutation in one of them with little co-occurrence between the three channels, suggesting that deregulation of any single channel isoform is sufficient to promote tumor growth. A previous study reported pharmacological inhibition of T-type calcium channels with mibefradil decreased medulloblastoma cell growth and invasion and increased cell death. Pharmacological inhibition of Group 3 and Group 4 medulloblastoma cell lines with mibefradil induced apoptosis and altered metabolic pathways [31]. Silencing of T-type calcium channels decreased cell growth and increased cell death demonstrating that the effect seen with mibefradil was specific to the inhibition of the T-type calcium channels.

To gain insight into the mechanism of T-type calcium channels in medulloblastoma we performed RPPA-based protein activation mapping of key signaling pathways and uncovered that mibefradil decreased expression of proliferative genes such as Ki67, CDK2, CDK6 and increased expression of apoptotic pathways genes BAD and BAX. We utilized publicly available single cell RNA-seq data to perform co-expression analysis to gain additional insight into mechanisms of T-type calcium channels in medulloblastoma. Co-expression analysis of T-type calcium channel in medulloblastoma tumors identified tumor modules representing numerous important biological processes associated with neuronal processes, cell signaling pathways, regulation of histone methylation, and cell division. We observed that modules for Cav3.1 and Cav3. typically trended in opposite directions. In the SHH tumors Cav3.1 had upregulation of SHH-Module 5 which was downregulated in Cav3.2 positive cells. Cav3.2 positive cells showed upregulation of SHH-Module 2 which was downregulated in Cav3.1 positive cells. This trend was also observed in the Group 3 tumors as well, suggesting that Cav3.1 and Cav3.2 regulate different cell states within the subgroups of medulloblastoma. These data demonstrate the T-type calcium channels are involved in various biological pathways in medulloblastoma tumors.

Mibefradil significantly reduced in vivo growth of medulloblastoma tumors and significantly prolonged animal survival. These data provide promising translational significance for the use of mibefradil in the treatment of medulloblastoma tumors. Mibefradil has been tested in clinical trials in recurrent glioblastoma patients in combination with temozolomide (NCT01480050) as well as in recurrent GBM patients with radiation (NCT02202993), which demonstrated that it was safe with some patients exhibiting partial responses [19, 32].

Altogether, this study represents the first fairly comprehensive analysis of the expression, functions, and mechanisms of action of T-type calcium channels in medulloblastoma. The study also indicates that blocking the channels with the repurposed FDA approved drug mibefradil is a promising potential therapy for one of the most common malignant pediatric brain tumors.

## Electronic supplementary material

Below is the link to the electronic supplementary material.

Supplementary Material 1: Fig. 1 Co-expression data for Group 3 tumors. (A) Dendogram of co-expression modules single Group 3 tumor cells. (B) kME for the Group 3 modules identified in hdWGCNA. (C) Gene ontology biological processes for each of the Group 3 modules. (D) Lollipop plot of the DME of Cav3.1 positive cells in Group 3 tumors. (E) Lollipop plot of the DME of Cav3.2 positive cells in Group 3 tumors.

Supplementary Material 2: Fig. 2 Co-expression data for SHH tumors. (A) Dendogram of co-expression modules single SHH tumor cells. (B) kME for the SHH modules identified in hdWGCNA. (C) Gene ontology biological processes for each of the SHH modules. (D) Lollipop plot of the DME of Cav3.1 positive cells in SHH tumors. (E) Lollipop plot of the DME of Cav3.2 positive cells in SHH tumors.

Supplementary Material 3: Fig. 3 Co-expression data for Group 4 tumors. (A) kME for the Group 4 modules identified in hdWGCNA. (B) Network plot of top 25 genes in Group 4-M1 module and Gene ontology biological processes for the module. (C) Network plot of top 25 genes in Group 4-M2 module and Gene ontology biological processes for the module. (D) Network plot of top 25 genes in Group 4-M3 module and Gene ontology biological processes for the module.

Supplementary Material 4: Fig. 4 Mibefradil and siRNA mediated silencing of T-type calcium channels induces cell death and inhibits cell growth. (A) PFSK cells were treated with vehicle or mibefradil (1–10 µM) and assessed for cell viability changes with Alamar Blue 48 h later. (B) PFSK cells were treated with vehicle or mibefradil and cell death was assessed 48 h later by trypan blue cell counting. (C) Cell growth assay of vehicle and mibefradil treated cell lines (PFSK). (D) qPCR and western blot confirmation of Cav3.2 knockdown in ONS-76 cells. (E) ONS-76 cells were transfected with Scrambled or siRNA for Cav3.2 and assessed for proliferation by cell counting over 7 days. (F) Cell death was assessed by trypan blue assay. *N* = 3 independent experiments for all assays *p < 0.05.

Supplementary Material 5: Fig. 5 Mibefradil significantly alters medulloblastoma signaling. (A) Heatmap summarizing the fold change of mibefradil treated ONS-76 and DAOY across the RPPA analytes that were screened. (B) Western Blot confirmation showing downregulation of CDK6, BAD and upregulation of BAK in mibefradil treated ONS-76 cells.

Supplementary Material 6

## Data Availability

No datasets were generated or analysed during the current study.
