# Supplementary material for: T-type calcium channels regulate medulloblastoma and can be targeted for therapy
Source: J Neurooncol. 2025 Feb 17;173(1):121–30. doi: 10.1007/s11060-025-04967-5 (PMC12041153; doi:10.1007/s11060-025-04967-5)
Supplement: Supplementary file 6 — Supplementary Material 6 [file 11060_2025_4967_MOESM6_ESM.docx]

**Supplemental Figure Legends:**

**Supplemental Figure 1:** Co-expression data for Group 3 tumors. A) Dendogram of co-expression modules single Group 3 tumor cells. B) kME for the Group 3 modules identified in hdWGCNA. C) Gene ontology biological processes for each of the Group 3 modules. D) Lollipop plot of the DME of Cav3.1 positive cells in Group 3 tumors. E) Lollipop plot of the DME of Cav3.2 positive cells in Group 3 tumors

**Supplemental Figure 2:** Co-expression data for SHH tumors. A) Dendogram of co-expression modules single SHH tumor cells. B) kME for the SHH modules identified in hdWGCNA. C) Gene ontology biological processes for each of the SHH modules. D) Lollipop plot of the DME of Cav3.1 positive cells in SHH tumors. E) Lollipop plot of the DME of Cav3.2 positive cells in SHH tumors

**Supplemental Figure 3:** Co-expression data for Group 4 tumors. A) kME for the Group 4 modules identified in hdWGCNA. B) Network plot of top 25 genes in Group 4-M1 module and Gene ontology biological processes for the module. C) Network plot of top 25 genes in Group 4-M2 module and Gene ontology biological processes for the module. D) Network plot of top 25 genes in Group 4-M3 module and Gene ontology biological processes for the module.

**Supplemental Figure 4:** Mibefradil and siRNA mediated silencing of T-type calcium channels induces cell death and inhibits cell growth. A) PFSK cells were treated with vehicle or mibefradil (1-10 µM) and assessed for cell viability changes with Alamar Blue 48 hours later. B) PFSK cells were treated with vehicle or mibefradil and cell death was assessed 48hrs later by trypan blue cell counting. C) Cell growth assay of vehicle and mibefradil treated cell lines (PFSK). D) qPCR and western blot confirmation of Cav3.2 knockdown in ONS-76 cells. E) ONS-76 cells were transfected with Scrambled or siRNA for Cav3.2 and assessed for proliferation by cell counting over 7 days. F) Cell death was assessed by trypan blue assay. N=3 independent experiments for all assays *p<0.05

**Supplemental Figure 5:** Mibefradil significantly alters medulloblastoma signaling. A) Heatmap summarizing the fold change of mibefradil treated ONS-76 and DAOY across the RPPA analytes that were screened. B) Western Blot confirmation showing downregulation of CDK6, BAD and upregulation of BAK in mibefradil treated ONS-76 cells.
